# Associations of Flavored e-Cigarette Uptake With Subsequent Smoking Initiation and Cessation

**DOI:** 10.1001/jamanetworkopen.2020.3826

**Published:** 2020-06-05

**Authors:** Abigail S. Friedman, SiQing Xu

**Affiliations:** 1 Department of Health Policy and Management, Yale School of Public Health, New Haven, Connecticut

## Abstract

**Importance:**

Several states have banned sales of flavored e-cigarettes, but evidence on the association between vaping flavors and subsequent smoking initiation and cessation is limited.

**Objective:**

To evaluate whether new uptake of flavored e-cigarettes is more strongly associated with subsequent smoking initiation and cessation than uptake of unflavored e-cigarettes, separately for youths (12-17 years), emerging adults (18-24 years), and prime-age adults (25-54 years).

**Design, Setting, and Participants:**

This cohort study conducted secondary data analyses of longitudinal survey data from waves 1 to 4 of the Population Assessment of Tobacco and Health Study (collected from 2013 to 2018). The analytic sample was limited to 17 929 respondents aged 12 to 54 years at wave 1 who completed at least 3 consecutive waves of the survey and did not use e-cigarettes at baseline. Data were collected from 2013 to 2018 and analyzed in February 2020.

**Exposures:**

Flavored vs unflavored e-cigarette use reported in wave 2 of the Population Assessment of Tobacco and Health Study.

**Main Outcomes and Measures:**

Binary indicators captured wave 3 smoking among 7311 youths and 4634 emerging adults who did not smoke at baseline (ie, initiation) and not smoking at wave 3 among 1503 emerging adults and 4481 prime-age adults who smoked at baseline (ie, cessation). Smoking status was based on having smoked in the past 30 days for youths and established smoking (ie, current smoking among those who smoked at least 100 cigarettes in their lifetime) for emerging and prime-age adults.

**Results:**

The youths who did not smoke at baseline, emerging adults who smoked at baseline, and prime-age adults who smoked at baseline consisted of 51.4% to 58.0% male participants and 66.9% to 77.0% white individuals. Vaping uptake was positively associated with smoking initiation in youth (adjusted odds ratio [AOR], 6.75; 95% CI, 3.93-11.57; *P* < .001) and in emerging adults (AOR, 3.20; 95% CI, 1.70-6.02; *P* < .001). Vaping uptake was associated with cessation in adults (AOR, 1.34; 95% CI, 1.02-1.75; *P* = .03). Vaping nontobacco flavors was no more associated with youth smoking initiation than vaping tobacco-flavors (AOR in youth, 0.66; 95% CI, 0.16-2.76; *P* = .56) but was associated with increased adult smoking cessation (AOR in adults, 2.28; 95% CI, 1.04-5.01; *P* = .04).

**Conclusions and Relevance:**

In this study, adults who began vaping nontobacco-flavored e-cigarettes were more likely to quit smoking than those who vaped tobacco flavors. More research is needed to establish the relationship between e-cigarette flavors and smoking and to guide related policy.

## Key Points

**Question:**

Does the association between vaping uptake and subsequent smoking differ between individuals favoring tobacco- vs nontobacco-flavored e-cigarettes?

**Findings:**

In this cohort study with 17 929 participants, multivariable analyses of nationally representative, longitudinal survey data evaluated differences in smoking initiation and cessation subsequent to vaping uptake among those who used flavored vs unflavored e-cigarettes, separately by age group. Relative to vaping tobacco flavors, vaping nontobacco-flavored e-cigarettes was not associated with increased youth smoking initiation but was associated with an increase in the odds of adult smoking cessation.

**Meaning:**

In this study, adults who vaped flavored e-cigarettes were more likely to subsequently quit smoking than those who used unflavored e-cigarettes.

## Teaser

This survey study evaluates whether new uptake of flavored e-cigarettes is more strongly associated with subsequent smoking initiation and cessation than uptake of unflavored e-cigarettes, separately for youths, emerging adults, and prime-age adults.

## Introduction

With increasing e-cigarette use, flavored e-cigarettes and their appeal to youths have become a prominent concern. Advocacy groups and the American Academy of Pediatrics emphasize that nontobacco flavors may motivate youth vaping (ie, e-cigarette use) and increase conventional cigarette use (smoking).^1,2,3^ Given these concerns, the US Food and Drug Administration announced that it will enforce sales restrictions on e-cigarette cartridges with flavors other than tobacco and menthol unless the product has obtained Food and Drug Administration premarket authorization. However, industry representatives claim that such flavors are critical to attracting adults who smoke and want to quit.^4,5,6^ The tension between these perspectives—nontobacco flavors as a risk to youth vaping initiation vs a boon for adult smoking cessation—remains unresolved. Because vaping’s effect on conventional smoking is central to its health influence, understanding how flavored e-cigarette use is related to smoking initiation and cessation is critical to guiding policy. Henceforth, flavored and unflavored e-cigarettes refer to nontobacco (eg, fruit, candy, menthol, mint) and tobacco flavors, respectively.

Randomized clinical trials show that e-cigarettes can aid in adult smoking cessation.^7,8,9,10,11^ These findings may apply to adolescents who smoke, although that evidence is less robust.^12^ Concurrently, a meta-analysis of research on e-cigarettes and youth smoking initiation finds “strong and consistent evidence of an association between initial e-cigarette use and subsequent cigarette smoking initiation.”^13^ A recent analysis using Population Assessment of Tobacco and Health Study data^2^ found that previous e-cigarette use was associated with a 4-fold increase in youths’ risk of ever using conventional cigarettes relative to youths who had not vaped.

The association between e-cigarette flavors and smoking is of particular interest. Qualitative evidence suggests young adults who smoke perceive flavors as helpful in cutting down conventional cigarette use.^14^ However, a cross-sectional analysis of middle and high school students who had never smoked found stronger intentions to try conventional cigarettes among those using flavored rather than unflavored e-cigarettes.^15^ Furthermore, new use of 1 tobacco product is more strongly associated with continued use of that product 1 year later for flavored rather than unflavored products.^16,17^ However, the association of flavors in one product with use of another remains unclear.

Bans on conventional cigarette flavors other than tobacco and menthol do not apply to e-cigarettes.^18^ In 2018, San Francisco banned sales of flavored tobacco products, including flavored e-cigarettes.^19^ In March 2019, Congresswoman Diana DiGette filed legislation to ban e-cigarette flavors that attract youths unless manufacturers proved they did not contribute to the increase in youth vaping.^20^ Michigan banned flavored e-cigarette sales that September, followed by New York and other states. Some of these bans have since been stayed by the courts. Similar legislation is under consideration at the federal level.

It remains unclear whether flavor bans benefit public health. Current evidence on how flavor bans affect smoking is limited to hypothetical choice experiments. These studies generally suggest that flavor options (beyond menthol and tobacco) affect both youth and adult consumers’ preferences for e-cigarettes.^21^ However, 1 study found that although interest in e-cigarettes among adults who smoke varied with flavor descriptors, interest among adolescents who do not smoke did not.^22^ A separate analysis of individuals aged 18 to 64 years who currently smoke or recently quit smoking concluded that a federal ban on e-cigarette flavors would increase smoking, whereas banning menthol conventional cigarettes would reduce smoking.^23^

To inform this debate, we used nationally representative, longitudinal data from the Population Assessment of Tobacco and Health Study to estimate the association between e-cigarette flavor choice and smoking initiation among those who did not smoke at baseline as well as cessation among those who did smoke at baseline, separately for youths (12-17 years), emerging adults (18-24 years), and prime-age adults (25-54 years). Previous research with these data suggests that vaping may contribute to youth smoking initiation.^2^ This article expands on that work not only by assessing how vaping uptake relates to smoking among emerging and prime-age adults and youths but also by evaluating whether these associations differ between those using flavored vs unflavored e-cigarettes. We hypothesized that vaping uptake would be associated with increased youth and emerging adult initiation as well as increased emerging and prime-age adult cessation but that these associations would not vary by flavored vs unflavored e-cigarette use.

## Methods

### Data

Analyses considered public-use data from waves 1 to 4 of the Population Assessment of Tobacco and Health Study. This longitudinal survey’s cohort was selected via a multistage, stratified probability sample, such that weighted analyses were nationally representative for the noninstitutionalized US civilian population.^24^ Wave 1 response rates were 75% and 78% for the youth and adult samples, respectively. Wave 3 response rates (within the wave 1 cohort) were 78% and 83%, respectively.^25^ Wave 1 was administered from September 2013 through December 2014, wave 2 from October 2014 to October 2015, wave 3 from October 2015 to October 2016, and wave 4 from December 2016 to January 2018. Alongside demographic characteristics, the Population Assessment of Tobacco and Health Study collected data on tobacco use and product characteristics, with separate youth (12-17 years) and adult (≥18 years) surveys. Responses were collected with audio computer-assisted self-interviewing in English or Spanish. Although not included in the public-use data, biospecimens related to tobacco exposure were collected from consenting nonminor respondents. This sample has been described in detail elsewhere.^26,27^ Yale University’s institutional review board deemed this study exempt from review, given the use of publicly available deidentified data. This study followed Strengthening the Reporting of Observational Studies in Epidemiology (STROBE) reporting guidelines.

### Study Population

Analyses required 3 consecutive waves of data to consider whether individuals who began vaping between waves 1 and 2 were more or less likely to either initiate or quit smoking—depending on their baseline smoking status—by wave 3. Stratifying by baseline smoking status yielded 4 analytic samples, all limited to those who did not vape at baseline, as follows: youths who did not smoke at baseline (aged 12-17 years; n = 7311), emerging adults who did not smoke at baseline (aged 18-24 years; n = 4634), emerging adults who smoked at baseline (n = 1503), and prime-age adults who smoked at baseline (aged 25-54 years; n = 4481) (eAppendix in the Supplement). Youth cessation and prime-age initiation were not considered because these events were rare in the data and may have been less likely to reflect true instances of quitting or new initiation (because of potential relapse).

### Outcome of Interest

Outcomes were binary indicators for self-reported smoking at wave 3 among those who did not smoke at wave 1 (ie, initiation) and reporting not smoking at wave 3 among those who did smoke at wave 1 (ie, cessation). To distinguish regular use from experimentation, adult smoking status was based on established smoking (ie, respondents who had smoked at least 100 cigarettes in their lifetime and currently smoked every day or some days). The youth survey did not ask about established smoking, so youth smoking status was based on recent smoking (ie, smoked in the past 30 days).

### Exposures

#### Vaping Status

With respondents who vaped at wave 1 omitted from the analytic sample, a binary indicator for wave 2 e-cigarette use was used to capture new vaping uptake. Including those who vaped at baseline could have biased results, because those who vaped for a long time may have had different smoking initiation and cessation patterns than those who recently started vaping. The public-use data did not report time since vaping initiation among those who vaped at wave 1. As with smoking, vaping indicators signified recent vaping (ie, past 30 days) for youths but were more consistent with established use for adults (ie, ever used an e-cigarette, have ever used fairly regularly, and currently use every day or some days).

#### Flavor Preferences

A categorical variable classified e-cigarette use as nontobacco flavored, tobacco flavored, or missing. For adults, this was based on a yes, no, or missing response regarding whether their “regular or last brand of e-cigarettes used was flavored to taste like menthol, mint, clove, spice, fruit, chocolate, alcoholic drinks, candy, or other sweets.” Youth flavor preferences were coded similarly; the only difference was that their survey asked about use in the past 30 days instead of regular or last brand used.

### Additional Controls

Given well-established differentials in smoking behavior by sex, age, race/ethnicity, income, and education, controls adjusted for these traits to ensure that basic demographic differences in e-cigarette product choice did not drive findings. Demographic controls were binary indicators for self-reported sex, age group (binned by Population Assessment of Tobacco and Health Study public-use data at 12-14, 15-17, 18-24, 25-34, 35-44, and 45-54 years), race (aggregated by the study’s public-use data to white, black, and other), and Hispanic ethnicity, with separate indicators for missing sex, race, and ethnicity observations.

Categorical income and education measures provided socioeconomic status controls. Household income observations came from wave 1 for adults and, because income was not reported in wave 1 public-use youth data, wave 2 for youths. Youth analyses controlled for wave 1 parental education (<high school, high school graduate or equivalent, some college or associate’s degree, college graduate or more, and missing). Adult analyses controlled for a binary respondent education indicator (completed any college) at wave 3 to avoid conflating completed with ongoing education.

Additional controls included an indicator for having ever tried cigarettes at baseline to account for baseline propensity to smoke in initiation analyses. Because favoring tobacco flavors might reflect an underlying desire to smoke, an additional control was considered to help adjust for selection bias in flavor-choice analyses, ie, a binary indicator for using e-cigarettes because “it feels like smoking a regular cigarette” at wave 2.

### Statistical Analysis

First, sample-weighted summary statistics characterized tobacco use and demographic characteristics by age group and wave 2 vaping status. Next, χ^2^ tests compared wave 3 smoking initiation and cessation by wave 2 vaping status and flavor choice for each age group. Finally, sample-weighted multivariable logistic regressions estimated how vaping uptake (between waves 1 and 2) was associated with smoking initiation and cessation by wave 3, and, limiting consideration to individuals who took up vaping, whether these associations differed by flavored vs unflavored e-cigarette use. All analyses adjusted for the aforementioned sociodemographic controls and, for initiation analyses, whether the respondent had ever tried conventional cigarettes at baseline.

For flavor analyses, specification checks added a control for respondents who cited that vaping feels like a cigarette as a reason they vape to help clarify whether estimated associations between flavored vs unflavored e-cigarette use and smoking were explained by selection bias in flavor choice. Sensitivity checks considered pooling emerging and prime-age adults to address small cessation analysis samples, using wave 4 smoking initiation or cessation as the outcome variable to assess longer-term relationships, and providing unweighted regressions for reference. Analyses were performed with Stata version 14.1 (StataCorp), applying *svy* commands to account for complex sample design, and reporting 2-tailed tests of statistical significance at the *P* < .05 level. Multiple imputation was not used because tobacco use nonresponse is unlikely to be missing at random.

## Results

### Summary Statistics and Cross-Tabulations

Among those who did not smoke at baseline, the analytic sample was 51.36% male individuals (95% CI, 50.01%-52.70%) and 66.91% white individuals (95% CI, 64.22%-69.48%) for youths (n = 7311) and 47.46% male individuals (95% CI, 45.96%-48.97%) and 66.51% white individuals (95% CI, 62.62%-68.30%) for emerging adults (n = 4634) (Table 1 [pooled results not shown]). Compared with those who did not vape at wave 2, those who took up vaping between waves 1 and 2 showed elevated rates of both having tried a conventional cigarette at baseline (youths: 4.86% [95% CI, 4.24%-5.57%] vs 22.77% [15.79%-31.67%]; *P* < .001; emerging adults: 40.03% [95% CI, 38.00%-42.10%] vs 67.52% [95% CI, 54.51%-78.29%]; *P* < .001) and smoking at wave 3 (youths: 2.84% [95% CI, 2.40%-3.35%] vs 21.80% [95% CI, 16.16%-28.73%]; *P* < .001; emerging adults: 5.34% [95% CI, 4.69%-6.06%] vs 21.91% [95% CI, 14.68%-31.39%]; *P* < .001) (Table 1). Comparing wave 3 smoking rates between those who did not smoke at baseline and vaped flavored vs unflavored e-cigarettes showed no statistically significant differences in smoking initiation by flavor choice.

**Table 1.** Summary Statistics for Those Who Did Not Smoke or Vape at Baseline, Among Youths and Emerging Adults^a^

Wave 2 vaping status	Vaped, % (95% CI)			
	No	Yes	Flavored	Unflavored
**Youths (12-17 y)**				
No.	7096	164	129	14
Ever tried cigarettes, wave 1	4.86 (4.24-5.57)	22.77 (15.79-31.67)	21.64 (14.64-30.78)	35.56 (10.63-71.91)
Smoked in past 30 d, wave 3	2.84 (2.40-3.35)	21.80 (16.16-28.73)	19.47 (13.82-26.71)	41.76 (14.80-74.74)
Male youths	51.18 (49.79-52.57)	56.69 (48.41-64.62)	57.19 (48.20-65.73)	42.83 (19.96-69.23)
Race				
White	66.88 (64.15-69.51)	74.56 (68.05-80.14)	72.36 (64.74-78.88)	88.68 (58.33-97.77)
Black	15.28 (13.27-17.53)	7.84 (4.57-13.14)	7.49 (3.64-14.79)	6.82 (0.75-41.53)
Other	13.68 (12.28-15.21)	13.53 (8.94-19.95)	16.32 (10.56-24.37)	0
Hispanic	22.40 (19.66-25.41)	20.64 (14.96-27.77)	19.95 (13.98-27.66)	23.39 (7.74-52.63)
Parental education				
<High school	17.18 (15.67-18.79)	18.78 (13.01-26.32)	20.11 (13.62-28.68)	20.94 (5.93-52.66)
High school graduate	17.35 (16.14-18.63)	19.58 (13.71-27.18)	18.43 (12.07-27.10)	29.31 (6.92-69.80)
Some college	30.79 (28.96-32.68)	30.60 (23.98-38.14)	34.14 (26.29-42.98)	4.69 (0.51-32.26)
≥College degree	34.17 (31.60-36.83)	31.04 (23.27-40.04)	27.32 (19.32-37.10)	45.07 (17.88-75.56)
Parental household income, $				
<10 000	7.23 (6.21-8.40)	7.76 (4.36-13.46)	7.53 (3.83-14.27)	10.71 (2.06-40.67)
10 000-24 999	13.86 (12.64-15.19)	15.76 (11.23-21.67)	16.13 (10.33-24.30)	21.29 (3.53-66.65)
25 000-49 999	20.02 (18.78-21.32)	17.99 (12.08-25.94)	14.65 (8.83-23.31)	29.99 (9.57-63.43)
50 000-99 999	23.41 (22.15-24.72)	28.54 (21.19-37.23)	28.11 (20.24-37.60)	21.46 (5.50-56.17)
≥100 000	23.61 (21.61-25.73)	21.82 (14.33-31.77)	23.79 (15.61-34.50)	16.55 (3.44-52.46)
**Emerging adults (18-24 y)**				
No.	4517	102	92	8
Ever tried cigarettes, wave 1	40.03 (38.00-42.10)	67.52 (54.51-78.29)	69.41 (56.08-80.13)	47.51 (11.97-85.77)
Established smoking, wave 3	5.34 (4.69-6.06)	21.91 (14.68-31.39)	23.79 (15.96-33.91)	8.17 (0.70-52.74)
Male emerging adults	47.00 (45.43-48.57)	65.73 (54.90-75.14)	62.68 (51.39-72.74)	93.84 (55.01-99.48)
Race				
White	65.44 (62.53-68.24)	71.25 (59.28-80.83)	70.48 (57.92-80.55)	74.59 (31.84-94.86)
Black	15.50 (13.46-17.79)	11.38 (6.29-19.71)	10.93 (5.76-19.77)	16.52 (2.49-60.57)
Other	15.36 (13.46-17.49)	13.51 (6.94-24.66)	14.22 (7.00-26.76)	8.89 (0.77-55.08)
Hispanic	21.82 (19.29-24.57)	23.22 (14.82-34.45)	25.18 (15.92-37.43)	8.89 (0.77-55.08)
Any college at baseline	59.40 (57.26-61.51)	39.77 (29.69-50.81)	42.26 (31.37-53.97)	17.07 (2.76-59.86)
Household income, $				
<10 000	24.17 (22.47-25.94)	22.85 (15.25-32.78)	24.48 (16.11-35.37)	0
10 000-24 999	20.14 (18.46-21.93)	23.87 (14.94-35.88)	24.64 (14.93-37.84)	19.94 (3.26-64.76)
25 000-49 999	16.89 (15.56-18.30)	14.82 (8.10-25.56)	13.13 (7.00-23.27)	31.14 (4.62-80.86)
50 000-99 999	15.40 (14.16-16.73)	12.83 (7.19-21.88)	13.59 (7.40-23.62)	7.77 (0.67-51.33)
≥100 000	10.64 (9.25-12.22)	12.04 (6.03-22.59)	8.83 (3.94-18.60)	41.15 (8.55-83.94)

^a^ Sample-weighted means use data from Population Assessment of Tobacco and Health Study waves 1 to 3. Age groups are based on age at wave 1. A total of 51 youths and 15 emerging adults lacked wave 2 vaping data. Among those who vaped at wave 2, flavor preference was missing for 14 youths and 2 emerging adults. Sex, race, Hispanic ethnicity, (parental) education, and (parental) income were missing in 0.26%, 4.18%, 2.33%, 0.50%, and 11.78% of youth observations, respectively; for emerging adults, the corresponding percentages were 0.9%, 3.68%, 0.45%, 0.44%, and 12.88%.

Emerging adults (n = 1503) and prime-age adults (n = 4481) who smoked at baseline were also primarily male and white individuals (male emerging adults: 57.97% [95% CI, 55.03%-60.86%]; male prime-age adults: 55.18% [53.48%-56.87%]; white emerging adults: 76.00% [95% CI, 72.92%-78.82%]; white prime-age adults: 76.96% [95% CI, 74.17%-79.53%]) (Table 2 [pooled results not shown]). Although prime-age adults who began vaping by wave 2 were more likely to quit smoking by wave 3 compared with those who did not vape (18.81% [95% CI, 14.55%-23.98%] vs 13.48% [95% CI, 12.21%-14.86%]; *P* = .02), this difference was not statistically significant for emerging adults. For both emerging and prime-age adults who took up vaping between waves 1 and 2, differences in smoking cessation rates by flavor choice were not statistically significant.

**Table 2.** Summary Statistics for Those Who Smoked and Did Not Vape at Baseline, Among Emerging and Prime-Age Adults^a^

Wave 2 vaping status	Vaped, % (95% CI)			
	No	Yes	Flavored	Unflavored
**Emerging adult (18-24 y)**				
No.	1343	158	128	23
Did not smoke, wave 3	20.11 (17.53-22.97)	23.55 (16.99-31.68)	26.86 (19.21-36.18)	12.53 (3.71-34.76)
Male emerging adults	57.72 (54.58-60.80)	60.14 (53.39-66.54)	56.72 (49.56-63.61)	77.44 (55.84-90.31)
Race				
White	75.33 (72.08-78.31)	81.97 (75.16-87.23)	81.35 (73.15-87.47)	82.95 (63.87-93.05)
Black	12.05 (9.71-14.85)	5.42 (2.66-10.70)	6.15 (2.90-12.57)	3.04 (0.37-20.90)
Other	10.13 (8.18-12.48)	10.41 (6.58-16.08)	9.78 (5.53-16.70)	14.01 (5.22-32.49)
Hispanic	14.82 (12.09-18.04)	13.33 (8.84-19.62)	14.39 (9.00-22.24)	11.50 (3.40-32.45)
Any college at baseline	45.69 (42.53-48.89)	48.24 (39.89-56.68)	48.57 (38.67-58.58)	39.00 (20.18-61.77)
Education at baseline missing	0.32 (0.11-0.93)	0.72 (0.10-5.04)	0.89 (0.12-6.14)	0
Household income, $				
<10 000	29.64 (26.98-32.45)	29.65 (22.02-38.62)	26.49 (18.98-35.67)	42.22 (22.97-64.16)
10 000-24 999	26.81 (24.16-29.63)	25.26 (19.21-32.45)	26.02 (19.41-33.93)	24.46 (10.50-47.18)
25 000-49 999	19.15 (16.67-21.89)	18.03 (12.85-24.71)	16.36 (10.98-23.69)	26.17 (13.04-45.58)
50 000-99 999	10.54 (8.84-12.52)	12.43 (7.87-19.07)	14.64 (9.09-22.75)	4.05 (0.50-26.20)
≥100 000	5.83 (4.33-7.82)	4.63 (2.33-8.99)	5.17 (2.53-10.25)	3.11 (0.38-21.26)
**Prime-age adult (25-54 y)**				
No.	4120	339	219	109
Did not smoke, wave 3	13.48 (12.21-14.86)	18.81 (14.55-23.98)	21.70 (16.46-28.05)	12.16 (6.45-21.75)
Male prime-age adults	54.96 (53.17-56.74)	57.69 (51.52-63.63)	54.75 (48.38-60.97)	60.70 (47.90-72.19)
Race				
White	76.17 (73.30-78.82)	86.05 (79.84-90.57)	83.95 (75.40-89.93)	92.65 (85.88-96.31)
Black	15.02 (12.68-17.70)	6.08 (3.48-10.43)	8.33 (4.52-14.85)	0.91 (0.12-6.57)
Other	7.40 (6.54-8.48)	7.29 (4.42-11.79)	7.45 (4.12-13.10)	5.23 (2.44-10.85)
Hispanic	12.38 (10.53-14.51)	5.96 (3.62-9.66)	6.77 (4.06-11.09)	3.43 (0.95-11.70)
Any college at baseline	44.71 (42.85-46.59)	52.42 (45.44-59.32)	55.34 (47.25-63.17)	46.04 (34.49-58.04)
Education at baseline missing	0.64 (0.36-1.14)	0.59 (0.13-2.65)	0.97 (0.21-4.29)	0
Household income, $				
<10 000	19.30 (17.72-20.99)	19.21 (15.05-24.19)	20.24 (14.73-27.16)	14.36 (8.99-22.16)
10 000-24 999	23.74 (22.14-25.41)	21.34 (16.60-27.01)	20.23 (14.52-27.47)	24.21 (15.86-35.11)
25 000-49 999	24.44 (23.01-25.92)	25.88 (21.39-30.94)	24.13 (18.56-30.74)	30.01 (21.02-40.85)
50 000-99 999	19.20 (17.62-20.87)	15.70 (11.73-20.70)	18.74 (13.20-25.90)	11.06 (6.78-17.53)
≥100 000	6.30 (5.41-7.32)	11.14 (7.48-16.28)	11.83 (7.71-17.73)	10.56 (4.78-21.74)

^a^ Sample-weighted means use data from Population Assessment of Tobacco and Health Study waves 1 to 3. Age groups are based on age at wave 1. Two emerging adults and 22 prime-age adults lacked wave 2 vaping data. Among individuals who vaped at wave 2, flavor preference was missing for 7 emerging and 11 prime-age adults. Race, Hispanic ethnicity, education, and income were missing in 2.47%, 0.53%, 0.36%, and 8.23% of emerging adult observations, respectively. For prime-age adults, percentage missing for wave 3 smoking status, sex, race, Hispanic ethnicity, education, and income were 0.12%, 0.06%, 1.36%, 1.53%, 0.63%, and 7.10%, respectively.

### Vaping, Smoking Initiation, and Smoking Cessation

**Figure 1.**
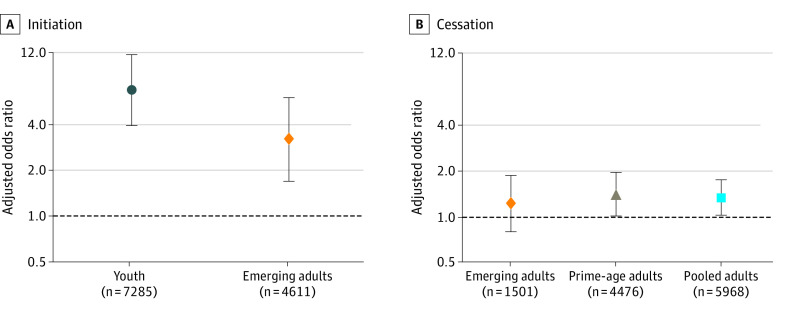
Adjusted Odds Ratios for the Association of Vaping Uptake With Subsequent Smoking Behavior Adjusted odds ratios and 95% CIs are presented from sample-weighted logistic regressions using data from waves 1 to 3 of the Population Assessment of Tobacco and Health Study. Vaping is defined as e-cigarette use in the past 30 days for youths and current established e-cigarette use for emerging and prime-age adults. Regressions controlled for fixed effects for male sex, race (black and other, with white as the reference group), Hispanic ethnicity, age group, household income categories (wave 2 parental reports for youths, wave 1 self-reports for adults), and (for initiation analyses only) an indicator for having ever tried conventional cigarettes at wave 1 as well as a missing-observation indicator for each of these variables. Additionally, youth regressions controlled for parental education at baseline (high school graduate or equivalent, some college, and ≥college graduate, with <high school graduate as the reference group), whereas adult regressions controlled for a binary indicator of completing any college to reflect adults’ own education at baseline. In youth and emerging adult initiation analyses, 19 and 4 respondents, respectively, with missing sex observations were omitted because the sex nonresponse indicator perfectly predicted initiation. Two respondents’ data were omitted from the prime-age and pooled adult cessation analyses for the same reason. The full regression output is available in eTable 1 and eTable 3 in the Supplement.

Figure 1 presents adjusted odds ratios (AORs) for the association of vaping uptake with smoking initiation or cessation by wave 3, along with corresponding 95% CIs. Consistent with previous research, new vaping was positively associated with smoking initiation by wave 3 for youths (AOR, 6.75; 95% CI, 3.93-11.57; *P* < .001) and emerging adults (AOR, 3.20; 95% CI, 1.70-6.02; *P* < .001). This association held for smoking initiation by wave 4 as well, with AORs of 5.62 for both youths (95% CI, 3.17-9.96; *P* < .001) and emerging adults (95% CI, 2.99-10.56; *P* < .001) (eTable 1 in the Supplement.) Unweighted analyses yielded similar implications (eTable 2 in the Supplement).

For individuals who smoked at baseline, vaping was associated with increased cessation among prime-age adults (AOR, 1.40; 95% CI, 1.01-1.96; *P* = .046). Although the AOR was not statistically significant for emerging adults (AOR, 1.22; 95% CI, 0.80-1.86; *P* = .36), it was significant in the pooled analyses for those aged 18 to 54 years (AOR, 1.34; 95% CI, 1.02-1.75; *P* = .03). Both findings became insignificant when wave 4 cessation was considered, although unweighted regressions yielded prime-age findings that were significant for cessation at both wave 3 (AOR, 1.49; 95% CI, 1.11-2.00; *P* = .01) and wave 4 (AOR, 1.38; 95% CI, 1.02-1.87; *P* = .04) (eTable 3 and eTable 4 in the Supplement).

### e-Cigarette Flavor Choice, Smoking Initiation, and Smoking Cessation

**Figure 2.**
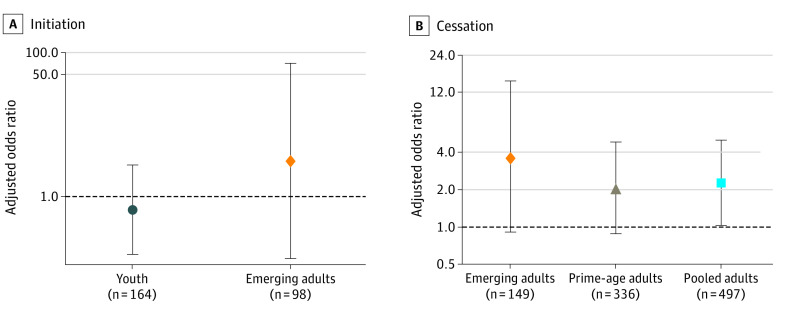
Adjusted Odds Rations for the Relative Association of Flavored vs Unflavored Vaping Uptake With Subsequent Smoking Adjusted odds ratios and 95% CIs are presented from sample-weighted logistic regressions using data from waves 1 to 3 of the Population Assessment of Tobacco and Health Study. Use of flavored vs unflavored e-cigarettes was attained from respondents categorized as vaping in wave 2. All regressions controlled for fixed effects for male sex, race (black and other, with white as the reference group), Hispanic, age group, household income categories (wave 2 parental reports for youths, wave 1 self-reports for adults), and (for initiation analyses only) an indicator for having ever tried conventional cigarettes at wave 1, as well as missing-observation indicators for each of these variables. Additionally, youth regressions controlled for baseline parental education (high school graduate or equivalent, some college, and ≥college graduate, with <high school graduate as the reference group), whereas adult regressions controlled for a binary indicator of completing any college to reflect adults’ own education at baseline. The emerging adult initiation analysis omitted data for 2 respondents with missing e-cigarette flavors, 1 respondent with missing Hispanic ethnicity, and 1 respondent with missing baseline education because of perfect predictivity. The emerging adult cessation analysis omitted data for 7 respondents with missing flavor responses, 1 with missing Hispanic ethnicity, and 1 with missing baseline education. The prime-age adult analysis omitted 3 responses with missing race. See eTables 5, 7, and 9 in the Supplement for full regression output.

Figure 2 presents AORs evaluating whether the association of e-cigarette use with subsequent smoking differed for flavored vs unflavored e-cigarettes. For both youths and emerging adults, the association of flavored e-cigarette use and smoking initiation was not significantly different from that for unflavored e-cigarette use (AOR for youth, 0.66; 95% CI, 0.16-2.76; *P* = .56; AOR for emerging adults, 3.15; 95% CI, 0.14-71.78; *P* = .46). Estimates remained statistically insignificant and moved slightly closer to 1 when controlling for whether respondents reported “it feels like a cigarette” as a reason for e-cigarette use (eTable 5 in the Supplement). However, this sensitivity check yielded a significant inverse association between flavored e-cigarette use and youth initiation by wave 4 (AOR, 0.25; 95% CI, 0.06-1.00; *P* = .049) (eTable 5 in the Supplement), with similar point estimates in unweighted analyses (eTable 6 in the Supplement).

For those who smoked at baseline, preferring flavored e-cigarettes had positive but statistically insignificant associations with emerging and prime-age adult cessation separately (eTable 7 and eTable 8 in the Supplement), but a significant association when these groups were pooled (AOR, 2.28; 95% CI, 1.04-5.01; *P* = .04) (eTable 9 in the Supplement). The latter estimate remained significant when adjusted for selection (AOR, 2.28; 95% CI, 1.04-4.99; *P* = .04) and in unweighted analyses (eTable 9 and eTable 10 in the Supplement).

## Discussion

This study’s findings support both sides of the current argument about the relationship between vaping and smoking: e-cigarette uptake is associated with increased youth and emerging adult smoking initiation but also increased cessation among prime-age adults who smoked at baseline. Comparing subsequent smoking behavior by uptake of flavored vs unflavored e-cigarettes yielded unexpected findings. Favoring flavored e-cigarettes was not associated with greater youth smoking initiation but was associated with greater adult smoking cessation; specifically, among adults who smoked and began vaping, the odds of cessation for those favoring nontobacco flavors were 2.3 times that of those who used tobacco-flavored e-cigarettes. Because early smoking cessation has substantial health benefits, with those who quit smoking before age 35 years experiencing a life expectancy similar to that of those who never smoked, increased cessation among individuals aged 18 to 54 years has substantive implications for population health.^28,29^

This study makes several contributions to the literature. To our knowledge, it constitutes the first analysis using nationally representative, longitudinal data to evaluate associations between e-cigarette flavor preferences and subsequent smoking behavior by age group, and thus provides critical evidence to inform the current policy debate. Additionally, by conducting analyses separately by age group, this work brings together 2 sets of literature that are often treated separately: the first, on vaping and youth smoking initiation; the second, on vaping and adult smoking cessation. Estimating these associations side by side allows a more comprehensive conversation about the relationship between e-cigarettes, smoking, and health, without privileging a single demographic group above another. Finally, this analysis distinguishes emerging and prime-age adults, 2 groups often evaluated as 1 but among whom smoking cessation may have very different implications for long-term health outcomes.

Critically, this study’s findings suggest that efforts to ban flavored e-cigarettes could increase smoking: nontobacco flavors were no more strongly associated with youth smoking initiation than tobacco flavors but were more strongly associated with adult cessation. Given limited sample sizes, further work is needed.

Nevertheless, these associations are not causal estimates. Certainly, some participants who began vaping would have initiated smoking regardless, and some participants who replaced traditional cigarettes with vaping would have quit even without e-cigarettes. However, it seems fair to say that the findings do not support the contention that flavored e-cigarette use is more strongly associated with minors’ subsequent smoking initiation than unflavored e-cigarette use and do support the argument that flavors are more strongly associated with smoking cessation among adults.

### Limitations

This study has several limitations, primarily related to the data. First, self-reported tobacco use may introduce social desirability bias. Absent access to the survey’s restricted biomarker data, this cannot be helped and may bias findings toward the null, although respondents’ knowledge that biomarkers were collected might have induced more accurate reporting. Second, because data collection for waves 1 and 2 largely preceded Juul’s introduction in 2015, the associations observed here may not generalize to nicotine salt e-cigarette products. Third, analyses cannot consider individuals who age out of the Population Assessment of Tobacco and Health Study’s youth samples, given differences between the youth and adult survey’s tobacco use questions and sample weights. This limits the youth and emerging adult analytic sample sizes, particularly reducing statistical power in analyses of wave 4 smoking behavior. Relatedly, analyses assessing differential relationships between flavored vs unflavored vaping and subsequent smoking are based on varying sample sizes, potentially explaining larger confidence intervals in some cases. Given the potential for overidentification in small sample analyses as well, further research with larger samples would be valuable.

Critically, this analysis does not establish a causal relationship between flavored e-cigarette use and smoking initiation or cessation. If individuals who want to quit are more likely to choose flavored e-cigarettes, this study’s results could stem from that initial preference. Randomized clinical trials are needed to clarify this relationship. Furthermore, in focusing on the association of vaping with smoking, we did not assess vaping’s health implications in the absence of smoking. More research is needed in that area.

## Conclusions

In this study, adults who began vaping nontobacco-flavored e-cigarettes were more likely to quit smoking than those who vaped tobacco flavors. This study’s findings are consistent with concerns about e-cigarettes’ influence on minors’ tobacco use and claims that flavored e-cigarettes help adults who smoke quit; specifically, evidence that adults who smoke and vape nontobacco flavors may be more likely to quit smoking than those using tobacco-flavored e-cigarettes suggests that banning flavors altogether may be too blunt an instrument for the current problem. Although proponents of flavor bans have claimed that tobacco-flavored e-cigarettes are adequate to help individuals who smoke, these results call for evidence to support that claim before it is acted on.^30^

## Notes

Supplement. **eAppendix. **Supplemental Methods **eTable 1.** Youth and Emerging Adult Initiation, Adjusted Odds Ratio/Standard Error **eTable 2. **Youth and Emerging Adult Initiation, Unweighted, Adjusted Odds Ratio/Standard Error **eTable 3.** Emerging and Prime Age Adult Cessation, Adjusted Odds Ratio/Standard Error **eTable 4.** Emerging and Prime-Age Adult Cessation, Unweighted, Adjusted Odds Ratio/Standard Error **eTable 5.** Youth and Emerging Adult Initiation by Flavor, Adjusted Odds Ratio/Standard Error **eTable 6.** Youth and Emerging Adult Initiation by Flavor, Unweighted, Adjusted Odds Ratio/Standard Error **eTable 7.** Emerging and Prime-Age Adult Cessation by Flavor, Adjusted Odds Ratio/Standard Error **eTable 8.** Emerging and Prime-Age Adult Cessation by Flavor, Unweighted, Adjusted Odds Ratio/Standard Error **eTable 9.** Adult Cessation by Flavor, Adjusted Odds Ratio/Standard Error **eTable 10.** Adult Cessation by Flavor, Unweighted, Adjusted Odds Ratio/Standard Error
