# Supplementary material for: Associations of Flavored e-Cigarette Uptake With Subsequent Smoking Initiation and Cessation
Source: JAMA Netw Open. 2020 Jun 5;3(6):e203826. doi: 10.1001/jamanetworkopen.2020.3826 (PMC7275248; doi:10.1001/jamanetworkopen.2020.3826)
Supplement: Supplement. — eAppendix. Supplemental Methods eTable 1. Youth and Emerging Adult Initiation, Adjusted Odds Ratio/Standard Error eTable 2. Youth and Emerging Adult Initiation, Unweighted, Adjusted Odds Ratio/Standard Error eTable 3. Emerging and Prime Age Adult Cessation, Adjusted Odds Ratio/Standard Error eTable 4. Emerging and Prime-Age Adult Cessation, Unweighted, Adjusted Odds Ratio/Standard Error eTable 5. Youth and Emerging Adult Initiation by Flavor, Adjusted Odds Ratio/Standard Error eTable 6. Youth and Emerging Adult Initiation by Flavor, Unweighted, Adjusted Odds Ratio/Standard Error eTable 7. Emerging and Prime-Age Adult Cessation by Flavor, Adjusted Odds Ratio/Standard Error eTable 8. Emerging and Prime-Age Adult Cessation by Flavor, Unweighted, Adjusted Odds Ratio/Standard Error eTable 9. Adult Cessation by Flavor, Adjusted Odds Ratio/Standard Error eTable 10. Adult Cessation by Flavor, Unweighted, Adjusted Odds Ratio/Standard Error [file jamanetwopen-3-e203826-s001.pdf]

Supplementary Online Content

Friedman AS, Xu SQ. Associations of flavored e-cigarette uptake with subsequent smoking initiation and cessation. *JAMA Netw Open*. 2020;3(6):e203826. doi:10.1001/jamanetworkopen.2020.3826

eAppendix. Supplemental Methods
eTable 1. Youth and Emerging Adult Initiation, Adjusted Odds Ratio/Standard Error
eTable 2. Youth and Emerging Adult Initiation, Unweighted, Adjusted Odds Ratio/Standard Error
eTable 3. Emerging and Prime Age Adult Cessation, Adjusted Odds Ratio/Standard Error
eTable 4. Emerging and Prime-Age Adult Cessation, Unweighted, Adjusted Odds Ratio/Standard Error
eTable 5. Youth and Emerging Adult Initiation by Flavor, Adjusted Odds Ratio/Standard Error
eTable 6. Youth and Emerging Adult Initiation by Flavor, Unweighted, Adjusted Odds Ratio/Standard Error
eTable 7. Emerging and Prime-Age Adult Cessation by Flavor, Adjusted Odds Ratio/Standard Error
eTable 8. Emerging and Prime-Age Adult Cessation by Flavor, Unweighted, Adjusted Odds Ratio/Standard Error
eTable 9. Adult Cessation by Flavor, Adjusted Odds Ratio/Standard Error
eTable 10. Adult Cessation by Flavor, Unweighted, Adjusted Odds Ratio/Standard Error

This supplementary material has been provided by the authors to give readers additional information about their work.

© 2020 Friedman AS et al. *JAMA Network Open.*

eAppendix. Supplemental Methods

To construct the study sample, all available Population Assessment of Tobacco and Health (PATH) survey waves (1-4) were merged together into one file by PERSONID (N=51,802). The sample was then limited to members of the "Wave 1 Cohort" (i.e., not the replenishment sample added at wave 4) who were interviewed at wave 1 ("baseline"): 13,648 youths (age 12-17), 9,110 emerging adults (age 18-24), and 16,114 prime age adults (25-54) at baseline.

For a respondent to be eligible for our main analytic sample, they must have completed the PATH survey at waves 1, 2, and 3, with all data coming from the same survey sample (i.e., using either the youth or adult instrument) due to differences in the tobacco use questions and weights. Based on this restriction, 44% of youths, 28% of emerging adults, and 26% of prime age adults did not qualify for the sample. Among the 13,648 baseline youths, 14% aged out of the youth survey by wave 2 (and completed the wave 2 adult survey) and 13% aged out between waves 2 and 3 (and completed the wave 3 adult survey). In total, 2,402 youths had incomplete information for reasons other than aging out of the youth survey. This left 7,595 eligible youths, 6,578 eligible emerging adults, and 11,959 eligible prime age adults.

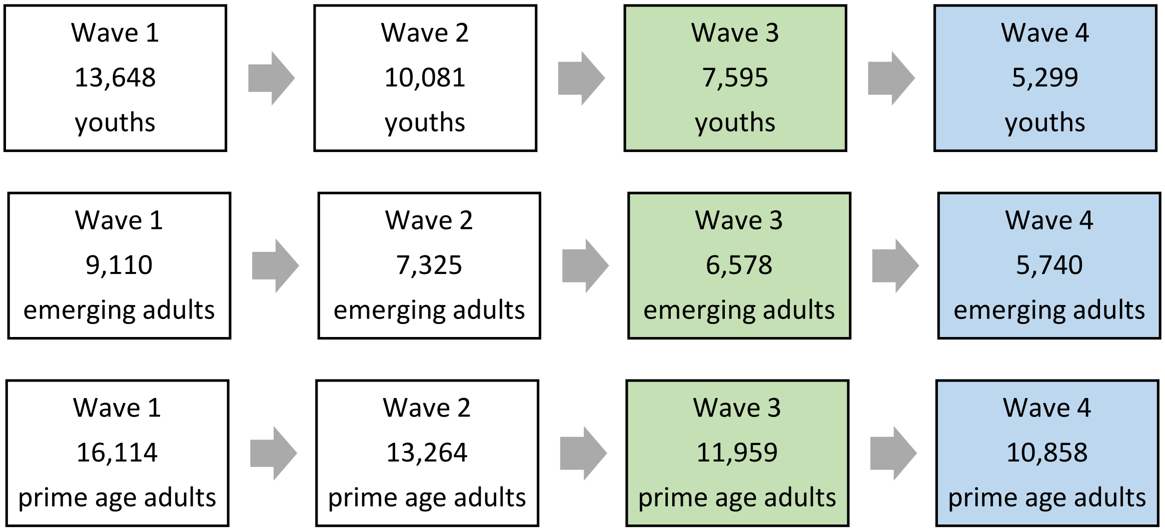

Final analytical samples consisted of 7,595 youths, 6,578 emerging adults, and 11,959 prime age adults, prior to exclusion of respondents who vaped at baseline. Using Stata's SVY suite and following the PATH user guidance, analyses were weighted by wave 3 longitudinal all-wave weights to account for PATH's complex survey design. Subpopulation analyses were considered for baseline non-vapers who were non-smokers, and baseline non-vapers who smoked, by wave 1 age-group (youth, emerging adult, or prime age adult). Taylor linearization methods were used to estimate standard errors.

The wave 4 sensitivity check used data on respondents who completed PATH surveys at all four waves. Among baseline respondents, 61%, 37%, and 33% of youths, emerging adults, and prime age adults respectively did not meet this requirement. (Note: some of these youths aged up into the adult PATH sample.) This left 5,299 youths, 5,740 emerging adults, and 10,858 prime age adults eligible for the sensitivity check samples. These analyses used wave 4 all-wave weights, consistent with PATH user guidance. Subpopulation analyses were defined as in the main analysis.

eTable 1. Youth and Emerging Adult Initiation, Adjusted Odds Ratio/Standard Error

	Initiation by Wave 3		Initiation by Wave 4	
	Youth [a]	Emerging Adult	Youth [a]	Emerging Adult
	(1)	(2)	(3)	(4)
Vaping Uptake[a]	6.7458***	3.1976***	5.6224***	5.6175***
	(1.836)	(1.019)	(1.620)	(1.788)
Ever tried cigarettes	5.9551***	9.8108***	6.1734***	8.0182***
	(1.262)	(1.844)	(1.296)	(1.466)
Male	0.6618**	1.0633	0.9619	1.2042
	(0.108)	(0.145)	(0.123)	(0.156)
**Race/Ethnicity**				
Hispanic	0.5454***	0.8829	0.6094**	0.8692
	(0.101)	(0.130)	(0.121)	(0.137)
Black	0.4274***	0.9376	0.2691***	0.8759
	(0.120)	(0.203)	(0.101)	(0.188)
Other race	0.7800	0.9361	0.7610	0.7535
	(0.186)	(0.168)	(0.155)	(0.139)
**Household Income**[b]				
$10,000 to $24,999	1.1742	0.6121**	0.9208	0.8510
	(0.317)	(0.115)	(0.255)	(0.167)
$25,000 to $49,999	0.9331	0.7199*	0.7009	0.8013
	(0.255)	(0.129)	(0.179)	(0.161)
$50,000 to $99,999	0.5649	0.7625	0.5159**	0.5950**
	(0.202)	(0.166)	(0.149)	(0.155)
$100,000 or more	0.4714**	0.4409***	0.4479***	0.5821*
	(0.160)	(0.122)	(0.129)	(0.160)
**Parental Education**				
HS or equivalent	0.9665		0.8767	
	(0.200)		(0.202)	
Some college	0.7804		0.9363	
	(0.141)		(0.174)	
College-plus	0.5189**		0.6838	
	(0.139)		(0.191)	
Any College		0.5839***		0.6485***
		(0.076)		(0.091)
15 to 17 years old	1.6805***		1.0657	
	(0.246)		(0.408)	
Constant	0.0574***	0.0244***	0.1005***	0.0273***
	(0.019)	(0.005)	(0.030)	(0.007)
N	7285	4611	5103	4129

Notes: Weighted logistic regressions use data on baseline non-smokers and non-vapers from waves 1-4 of the PATH youth and adult samples. All controls are listed except binary non-response indicators for each of the above variables. As some missing indicators perfectly predicted initiation, specifications 1, 2, 3, and 4 drop 19, 4, 17, and 3 respondents, respectively, with missing gender responses. Specifications 2 and 4 also drop 19 and 18 respondents with missing baseline education, respectively. Finally, specification 3 drops 29 respondents with missing parental education at baseline *(**)*** denote significance at the 0.1(0.05)0.01 levels. Linearized standard errors are adjusted for complex survey structure. Strata with a single sampling unit are treated as certainty units.

[a] For the youth and emerging adult samples, vaping and smoking initiation reflect past-30-day use and established use, respectively.

[b] Household income is reported in wave 2 parental interviews for youths and self-reported by emerging adults at baseline.

© 2020 Friedman AS et al. *JAMA Network Open.*

eTable 2. Youth and Emerging Adult Initiation, Unweighted, Adjusted Odds Ratio/Standard Error

	**Initiation by Wave 3**		**Initiation by Wave 4**	
	Youth [a]	Emerging Adult	Youth [a]	Emerging Adult
	(1)	(2)	(3)	(4)
Vaping Uptake[a]	6.6761***	2.3482***	5.9246***	3.3739***
	(1.520)	(0.601)	(1.523)	(0.836)
Ever tried cigarettes	6.6459***	9.5886***	6.3988***	8.3679***
	(1.089)	(1.707)	(1.201)	(1.403)
Male	0.6986**	1.0821	1.0355	1.1370
	(0.100)	(0.126)	(0.144)	(0.132)
**Race/Ethnicity**				
Hispanic	0.5818***	0.7964	0.6389**	0.7930
	(0.107)	(0.113)	(0.113)	(0.112)
Black	0.5161***	0.8231	0.2668***	0.8143
	(0.121)	(0.132)	(0.074)	(0.129)
Other race	0.7638	1.0768	0.7778	0.9495
	(0.156)	(0.181)	(0.154)	(0.162)
**Household Income**[b]				
$10,000 to $24,999	1.0322	0.6338***	0.8193	0.7738
	(0.265)	(0.104)	(0.206)	(0.123)
$25,000 to $49,999	0.8451	0.7530	0.5730**	0.7443
	(0.213)	(0.135)	(0.146)	(0.136)
$50,000 to $99,999	0.5508**	0.7077*	0.4104***	0.6023**
	(0.153)	(0.138)	(0.111)	(0.125)
$100,000 or more	0.4446**	0.4416***	0.3932***	0.5249**
	(0.142)	(0.117)	(0.118)	(0.138)
**Parental Education**				
HS or equivalent	0.9104		1.0155	
	(0.193)		(0.216)	
Some college	0.8633		1.1031	
	(0.171)		(0.218)	
College-plus	0.5737**		0.7052	
	(0.144)		(0.171)	
Any College		0.5368***		0.5450***
		(0.065)		(0.066)
15 to 17 years old	1.7419***		1.6519	
	(0.257)		(0.518)	
Constant	0.0512***	0.0300***	0.0955***	0.0366***
	(0.014)	(0.006)	(0.025)	(0.007)
N	7285	4611	5103	4129

Notes: Unweighted logistic regressions use data on baseline non-smokers and non-vapers from waves 1-4 of the PATH youth and adult samples. All controls are listed except binary non-response indicators for each of the above variables. As some missing indicators perfectly predicted initiation, specifications 1, 2, 3, and 4 drop 19, 4, 17, and 3 respondents, respectively, with missing gender responses. Specifications 2 and 4 also drop 19 and 18 respondents with missing baseline education, respectively. Finally, specification 3 drops 29 respondents with missing parental education at baseline. *(**)*** denote significance at the 0.1(0.05)0.01 levels.

[a] For the youth and emerging adult samples, vaping and smoking initiation reflect past-30-day use and established use, respectively.

[b] Household income is reported in wave 2 parental interviews for youths and self-reported by emerging adults at baseline.

© 2020 Friedman AS et al. *JAMA Network Open.*

eTable 3. Emerging and Prime-Age Adult Cessation, Adjusted Odds Ratio/Standard Error

	Cessation by Wave 3			Cessation by Wave 4		
	Emerging Adults	Prime Age Adults	Adults age 18-54	Emerging Adults	Prime Age Adults	Adults age 18-54
	(1)	(2)	(3)	(4)	(5)	(6)
Vaping Uptake	1.2187	1.4031**	1.3392**	0.7845	1.3110	1.1624
	(0.261)	(0.235)	(0.182)	(0.207)	(0.237)	(0.166)
Male	1.3093*	1.1475	1.1742**	1.1407	1.1778	1.1634
	(0.192)	(0.107)	(0.095)	(0.189)	(0.132)	(0.114)
**Race/Ethnicity**						
Hispanic	1.0261	1.8081***	1.5634***	1.5763**	1.3479**	1.3797***
	(0.186)	(0.253)	(0.184)	(0.290)	(0.192)	(0.164)
Black	1.0472	0.9705	0.9741	1.3150	0.9799	1.0297
	(0.235)	(0.147)	(0.116)	(0.317)	(0.156)	(0.133)
Other race	0.9793	1.6931***	1.4622**	0.4988**	1.5818**	1.2242
	(0.337)	(0.335)	(0.264)	(0.148)	(0.328)	(0.216)
**Household Income**						
$10,000 to $24,999	1.0466	1.5317**	1.3216**	1.1600	1.2934	1.2267
	(0.191)	(0.254)	(0.149)	(0.255)	(0.244)	(0.164)
$25,000 to $49,999	1.0953	1.7993***	1.4904***	1.3838	1.8012***	1.6345***
	(0.230)	(0.286)	(0.194)	(0.326)	(0.302)	(0.216)
$50,000 to $99,999	1.0777	2.5557***	1.9986***	1.2354	2.0390***	1.7987***
	(0.306)	(0.491)	(0.308)	(0.359)	(0.393)	(0.290)
$100,000 or more	1.2973	3.9924***	2.9131***	1.5941	2.9899***	2.5233***
	(0.494)	(0.740)	(0.468)	(0.635)	(0.641)	(0.452)
Any College	2.4397***	1.7087***	1.8873***	2.2350***	1.9533***	2.0360***
	(0.417)	(0.159)	(0.162)	(0.349)	(0.188)	(0.162)
**Baseline Age**						
25 to 34 years old			0.7245***			0.7901**
			(0.081)			(0.083)
35 to 44 years old		0.7474**	0.5490***		0.6805***	0.5426***
		(0.086)	(0.063)		(0.075)	(0.062)
45 to 54 years old		0.6736***	0.4902***		0.7359**	0.5759***
		(0.087)	(0.062)		(0.099)	(0.069)
Constant	0.1236***	0.0635***	0.1017***	0.1466***	0.0857***	0.1200***
	(0.019)	(0.010)	(0.012)	(0.028)	(0.015)	(0.016)
N	1501	4476	5978	1290	4015	5307

Notes: Weighted logistic regressions use data on baseline smokers and non-vapers from waves 1-4 of the PATH adult samples. All controls are listed except binary non-response indicators for each of the above variables. Vaping and smoking are defined as binary indicators for current established use. Specifications 1 and 4, drop 1 and 2 respondents who do not report vaping status at wave 2, respectively, as it perfectly predicted the outcome. Specifications 2 and 5 each drop 2 respondents with missing gender, as missing gender perfectly predicted the outcome. Specifications 3 and 6 drop 2 respondents with missing gender. *(**)*** denote significance at the 0.1(0.05)0.01 levels. Linearized standard errors are adjusted for complex survey structure. Strata with a single sampling unit are treated as certainty units.

© 2020 Friedman AS et al. *JAMA Network Open.*

eTable 4. Emerging and Prime-Age Adult Cessation, Unweighted, Adjusted Odds Ratio/Standard Error

	Cessation by Wave 3			Cessation by Wave 4		
	Emerging Adults	Prime Age Adults	Adults age 18-54	Emerging Adults	Prime Age Adults	Adults age 18-54
	(1)	(2)	(3)	(4)	(5)	(6)
Vaping Uptake	1.1712	1.4902***	1.3467**	0.7872	1.3823**	1.1508
	(0.250)	(0.225)	(0.166)	(0.194)	(0.212)	(0.150)
Male	1.2931*	1.1880*	1.2130**	1.2811*	1.1779*	1.1997**
	(0.181)	(0.108)	(0.092)	(0.186)	(0.106)	(0.091)
**Race/Ethnicity**						
Hispanic	1.1274	1.9308***	1.6049***	1.5622**	1.5485***	1.5339***
	(0.216)	(0.240)	(0.167)	(0.298)	(0.201)	(0.164)
Black	1.1683	1.0225	1.0500	1.4338*	1.0332	1.1184
	(0.234)	(0.144)	(0.120)	(0.289)	(0.140)	(0.124)
Other race	0.7561	1.5561***	1.2140	0.5179**	1.4602***	1.0702
	(0.172)	(0.224)	(0.147)	(0.136)	(0.214)	(0.136)
**Household Income**						
$10,000 to $24,999	1.0495	1.5769***	1.2876**	1.1191	1.3100*	1.1992
	(0.191)	(0.248)	(0.150)	(0.216)	(0.196)	(0.140)
$25,000 to $49,999	1.0670	1.8964***	1.4705***	1.3732	1.7430***	1.5601***
	(0.218)	(0.295)	(0.175)	(0.291)	(0.255)	(0.184)
$50,000 to $99,999	1.0235	2.5355***	1.8360***	1.1194	2.0002***	1.6843***
	(0.258)	(0.415)	(0.237)	(0.296)	(0.315)	(0.219)
$100,000 or more	1.4575	3.9211***	2.7279***	1.6140	3.0194***	2.4930***
	(0.458)	(0.770)	(0.436)	(0.545)	(0.583)	(0.407)
Any College	2.1597***	1.6461***	1.8230***	2.1122***	2.0034***	2.0667***
	(0.304)	(0.159)	(0.145)	(0.311)	(0.192)	(0.165)
**Baseline Age**						
25 to 34 years old			0.7569***			0.7838**
			(0.074)			(0.078)
35 to 44 years old		0.8128*	0.6254***		0.7891**	0.6276***
		(0.086)	(0.067)		(0.084)	(0.068)
45 to 54 years old		0.6901***	0.5193***		0.7478***	0.5797***
		(0.077)	(0.058)		(0.081)	(0.064)
Constant	0.1237***	0.0598***	0.0980***	0.1311***	0.0790***	0.1130***
	(0.021)	(0.009)	(0.012)	(0.023)	(0.012)	(0.014)
N	1501	4476	5978	1290	4015	5307

Notes: Unweighted logistic regressions use data on baseline smokers and non-vapers from waves 1-4 of the PATH adult samples. All controls are listed except binary non-response indicators for each of the above variables. Vaping and smoking are defined as binary indicators for current established use. Specifications 1 and 3, drop 1 and 2 respondents who do not report vaping status at wave 2, respectively, as it perfectly predicted the outcome. Specifications 2 and 5 each drop 2 respondents with missing gender, as missing gender perfectly predicted the outcome. Specifications 3 and 6 drop 2 respondents with missing gender. *(**)*** denote significance at the 0.1(0.05)0.01 levels.

© 2020 Friedman AS et al. *JAMA Network Open.*

eTable 5. Youth and Emerging Adult Initiation by Flavor, Adjusted Odds Ratio/Standard Error

	Initiation by Wave 3				Initiation by Wave 4			
	Youth [a]		Emerging Adults		Youth [a]		Emerging Adults	
	(1)	(2)	(3)	(4)	(5)	(6)	(7)	(8)
Vapes non-tobacco flavors[a]	0.6550	0.7280	3.1542	2.9822	0.2523*	0.2451**		
	(0.469)	(0.574)	(4.928)	(4.453)	(0.182)	(0.168)		
Ever tried cigarettes	2.0364	1.9206	1.7865	1.6709	1.6648	1.6192	1.4558	0.9280
	(1.041)	(0.994)	(1.166)	(1.138)	(0.814)	(0.800)	(0.882)	(0.659)
Use e-cigarettes because they		1.4389		1.4703		0.8560		7.3342***
feel like cigarettes		(0.826)		(0.751)		(0.375)		(5.380)
Male	0.7546	0.7978	1.8358	1.9070	1.0481	1.0485	1.5470	1.8426
	(0.376)	(0.391)	(1.107)	(1.160)	(0.461)	(0.463)	(0.909)	(1.325)
Hispanic ethnicity	0.7548	0.9038	0.3597	0.3492	0.4644**	0.4805*	0.1992**	0.1522**
	(0.505)	(0.644)	(0.276)	(0.275)	(0.165)	(0.181)	(0.133)	(0.117)
Race: Black	0.3520	0.3216	0.9676	0.9646	1.2721	1.4790	0.5468	0.4889
	(0.285)	(0.266)	(0.810)	(0.823)	(1.394)	(1.526)	(0.396)	(0.439)
Race: Other	0.4395	0.4084	0.5565	0.6123	1.7252	1.7088	0.6332	0.7397
	(0.325)	(0.313)	(0.496)	(0.556)	(0.854)	(0.859)	(0.533)	(0.685)
**Household Income**[b]								
$10,000 to $24,999	1.6265	1.5197	0.4018	0.3588	6.2125***	6.4604***	0.3471*	0.1706**
	(0.996)	(0.928)	(0.298)	(0.285)	(3.689)	(3.769)	(0.213)	(0.124)
$25,000 to $49,999	1.0978	0.9601	0.3672	0.3471	3.8005*	3.8798*	0.4382	0.2870
	(0.725)	(0.611)	(0.318)	(0.300)	(2.731)	(2.726)	(0.408)	(0.248)
$50,000 to $99,999	0.3547*	0.3155*	4.5596**	4.3687*	2.5834	2.8479	1.6476	1.1971
	(0.216)	(0.201)	(3.358)	(3.289)	(1.845)	(1.886)	(1.595)	(1.088)
$100,000 or more	0.3964	0.3826*	0.2744	0.2675	4.8880**	5.1597**	0.2058	0.3530
	(0.221)	(0.201)	(0.313)	(0.300)	(3.666)	(3.753)	(0.317)	(0.546)
Parental Education: HS or	1.5757	1.8093			0.7271	0.7224		
equivalent	(1.079)	(1.277)			(0.555)	(0.508)		
Parental Education: Some	0.6725	0.7244			0.8465	0.7896		
college	(0.485)	(0.539)			(0.550)	(0.469)		
Parental Education: College-plus	1.6951	2.0108			0.5248	0.4712		
	(1.083)	(1.265)			(0.397)	(0.325)		
Any College			1.0513	1.0864			1.3288	2.0159
			(0.659)	(0.691)			(0.876)	(1.452)
15 to 17 years old	0.6911	0.6977						
	(0.317)	(0.319)						
Constant	0.6922	0.5505	0.0692*	0.0652*	0.3654	0.3936	0.8500	0.5350

© 2020 Friedman AS et al. *JAMA Network Open.*

	(0.477)	(0.452)	(0.110)	(0.098)	(0.282)	(0.289)	(0.717)	(0.444)
N	164	161	98	98	93	92	81	81

Notes: Weighted logistic regressions use data on baseline non-smokers and non-vapers from waves 1-4 of the PATH youth and adult samples. All controls are listed except binary non-response indicators for each of the above variables. Some observations with missing responses are dropped as they perfectly predicted initiation. Specification 2 drops 3 respondents with missing responses about whether or not they use e-cigarettes because they feel like cigarettes. Specifications 3 and 4 drop 2 respondents with missing e-cigarette flavors, 1 respondent with missing Hispanic ethnicity, and 1 respondent with missing baseline education. Specifications 5 and 6 drop 5 respondents with missing Hispanic ethnicity, 2 respondents with missing baseline age; furthermore, baseline age was omitted due to collinearity. Specification 6 also drops 1 respondent with a missing response about whether or not they use e-cigarettes because they feel like cigarettes. Specifications 7 and 8 each drop 1 respondent with missing baseline education and drops the flavor variables as they perfectly predict initiation. *(**)*** denote significance at the 0.1(0.05)0.01 levels. Linearized standard errors are adjusted for complex survey structure. Strata with a single sampling unit are treated as certainty units.

[a] For the youth and emerging adult samples, vaping and smoking initiation reflect past-30-day use and established use, respectively.

[b] Household income is reported in wave 2 parental interviews for youths and self-reported by emerging adults at baseline.

© 2020 Friedman AS et al. *JAMA Network Open.*

eTable 6. Youth and Emerging Adult Initiation by Flavor, Unweighted, Adjusted Odds Ratio/Standard Error

	Initiation by Wave 3				Initiation by Wave 4			
	Youth [a]		Emerging Adults		Youth [a]		Emerging Adults	
	(1)	(2)	(3)	(4)	(5)	(6)	(7)	(8)
Vapes non-tobacco flavors[a]	0.7385	0.7986	2.0364	1.9711	0.2003*	0.1792*		
	(0.520)	(0.572)	(2.561)	(2.498)	(0.193)	(0.176)		
Ever tried cigarettes	2.9407**	2.8283**	1.6075	1.5471	2.3202	2.1847	1.4606	1.2472
	(1.354)	(1.306)	(1.083)	(1.065)	(1.464)	(1.394)	(1.033)	(0.916)
Use e-cigarettes because they feel like cigarettes		1.2258		2.1596		0.5986		4.4634**
		(0.659)		(1.183)		(0.396)		(2.709)
Male	0.8149	0.8522	1.4568	1.5191	1.1029	1.0900	1.8270	1.9831
	(0.359)	(0.377)	(0.824)	(0.868)	(0.594)	(0.586)	(1.073)	(1.243)
Hispanic ethnicity	0.7752	0.9099	0.4620	0.4550	0.3814	0.3606	0.3358	0.3285
	(0.422)	(0.523)	(0.308)	(0.315)	(0.247)	(0.245)	(0.229)	(0.244)
Race: Black	0.7565	0.7190	0.6216	0.5600	1.9508	2.7639	0.8777	0.7373
	(0.680)	(0.654)	(0.498)	(0.460)	(2.995)	(4.289)	(0.657)	(0.589)
Race: Other	0.4653	0.4456	0.7729	0.8433	1.8353	1.8613	0.7228	0.8195
	(0.303)	(0.291)	(0.617)	(0.681)	(1.319)	(1.343)	(0.566)	(0.667)
**Household Income**[b]								
$10,000 to $24,999	1.8181	1.7664	0.5403	0.4922	8.2477	8.3949	0.3004	0.2431*
	(1.551)	(1.515)	(0.401)	(0.375)	(11.656)	(11.581)	(0.230)	(0.203)
$25,000 to $49,999	1.1972	1.1130	0.6716	0.6009	5.1514	5.0412	0.5678	0.4262
	(1.082)	(1.020)	(0.552)	(0.502)	(7.186)	(6.899)	(0.443)	(0.349)
$50,000 to $99,999	0.5796	0.5686	2.9131	2.8014	4.4887	5.0479	1.4851	1.3240
	(0.535)	(0.536)	(2.383)	(2.312)	(6.634)	(7.342)	(1.279)	(1.168)
$100,000 or more	0.5870	0.5930	0.5295	0.5101	6.4904	6.6485	0.5583	0.9320
	(0.598)	(0.606)	(0.674)	(0.672)	(9.989)	(10.037)	(0.709)	(1.223)
Parental Education: HS or equivalent	1.0792	1.2021			0.3874	0.3579		
	(0.734)	(0.839)			(0.363)	(0.344)		
Parental Education: Some college	0.4874	0.5132			0.7580	0.6523		
	(0.342)	(0.362)			(0.656)	(0.580)		
Parental Education: College-plus	1.2930	1.4597			0.5744	0.4404		
	(0.973)	(1.145)			(0.560)	(0.453)		
Any College			1.7769	2.1352			1.6637	2.6678
			(0.977)	(1.224)			(0.977)	(1.727)
15 to 17 years old	0.7716	0.7851						
	(0.348)	(0.355)						
Constant	0.4825	0.3989	0.0927	0.0686	0.3334	0.4753	0.3989	0.2163*

© 2020 Friedman AS et al. *JAMA Network Open.*

	(0.469)	(0.407)	(0.145)	(0.112)	(0.491)	(0.726)	(0.335)	(0.198)
N	164	161	98	98	93	92	81	81

Notes: Unweighted logistic regressions use data on baseline non-smokers and non-vapers from waves 1-4 of the PATH youth and adult samples. All controls are listed except binary non-response indicators for each of the above variables. Some observations with missing responses are dropped as they perfectly predicted initiation. Specification 2 drops 3 respondents with missing responses about whether or not they use e-cigarettes because they feel like cigarettes. Specifications 3 and 4 drop 2 respondents with missing e-cigarette flavors, 1 respondent with missing Hispanic ethnicity, and 1 respondent with missing baseline education. Specifications 5 and 6 drop 5 respondents with missing Hispanic ethnicity, 2 respondents with missing baseline age; furthermore, baseline age was omitted due to collinearity. Specification 6 also drops 1 respondent with a missing response about whether or not they use e-cigarettes because they feel like cigarettes. Specifications 7 and 8 each drop 1 respondent with missing baseline education and drops the flavor variables as they perfectly predict initiation. *(**)*** denote significance at the 0.1(0.05)0.01 levels.

[a] For the youth and emerging adult samples, vaping and smoking initiation reflect past-30-day use and established use, respectively.

[b] Household income is reported in wave 2 parental interviews for youths and self-reported by emerging adults at baseline.

© 2020 Friedman AS et al. *JAMA Network Open.*

eTable 7. Emerging and Prime-Age Adult Cessation by Flavor, Adjusted Odds Ratio/Standard Error

	**Cessation by Wave 3**				**Cessation by Wave 4**			
	Emerging Adults		Prime Age Adults		Emerging Adults		Prime Age Adults	
	(1)	(2)	(3)	(4)	(5)	(6)	(7)	(8)
Vapes non-tobacco flavors	3.7047*	3.9205*	2.0732*	2.0798*	2.5894	2.5803	2.0127*	2.0087*
	(2.582)	(2.945)	(0.888)	(0.882)	(2.625)	(2.757)	(0.826)	(0.829)
Use e-cigarettes because		1.6372		0.8700		1.9437		1.0697
they feel like cigarettes		(0.864)		(0.309)		(1.101)		(0.367)
Male	4.5629***	4.9273***	2.1643**	2.1721**	2.1752	2.4394	1.9336*	1.9274*
	(2.218)	(2.365)	(0.665)	(0.676)	(1.697)	(1.955)	(0.699)	(0.708)
**Race/Ethnicity**								
Hispanic	0.3645	0.3214	1.1591	1.1504	1.9614	1.6297	1.4709	1.4751
	(0.257)	(0.226)	(0.836)	(0.818)	(1.667)	(1.398)	(1.030)	(1.036)
Black	1.1149	1.0707	0.6997	0.6802	2.9418	3.1269	0.6388	0.6424
	(1.314)	(1.190)	(0.596)	(0.599)	(3.631)	(3.717)	(0.580)	(0.589)
Other race	0.7689	0.7591	2.1819	2.1386	0.0963*	0.0710*	1.0795	1.0834
	(0.718)	(0.713)	(1.131)	(1.118)	(0.113)	(0.097)	(0.740)	(0.746)
**Household Income**								
$10,000 to $24,999	0.7688	0.7870	1.9637	1.9869	3.2155	3.7051	2.9242**	2.8872**
	(0.544)	(0.596)	(0.898)	(0.876)	(3.959)	(4.170)	(1.500)	(1.455)
$25,000 to $49,999	0.5592	0.6052	0.8841	0.8936	12.2887**	14.3006***	1.4572	1.4405
	(0.374)	(0.425)	(0.381)	(0.373)	(12.266)	(12.685)	(0.744)	(0.735)
$50,000 to $99,999	0.5064	0.5024	1.5781	1.5941	3.7877	3.9642	1.2518	1.2387
	(0.414)	(0.416)	(0.875)	(0.892)	(5.064)	(4.844)	(0.751)	(0.743)
$100,000 or more	1.8714	1.9758	3.2596**	3.3441**	34.7958***	44.1281***	3.9299**	3.8639**
	(1.639)	(1.838)	(1.906)	(1.923)	(46.017)	(54.797)	(2.592)	(2.536)
Any College	11.8541***	11.9413***	3.3236***	3.2803***	8.0332**	8.0361***	2.5280***	2.5316***
	(6.589)	(6.954)	(1.305)	(1.286)	(6.513)	(6.285)	(0.794)	(0.800)
**Baseline Age**								
35 to 44 years old			0.9452	0.9575			0.9867	0.9808
			(0.345)	(0.351)			(0.316)	(0.316)
45 to 54 years old			1.1434	1.1645			1.5493	1.5323
			(0.579)	(0.611)			(0.758)	(0.783)
Constant	0.0123***	0.0091***	0.0242***	0.0258***	0.0040***	0.0026***	0.0352***	0.0345***
	(0.010)	(0.008)	(0.016)	(0.017)	(0.005)	(0.004)	(0.024)	(0.024)
N	149	149	336	335	125	125	281	281

Notes: Weighted logistic regressions use data from waves 1-4 of the PATH adult samples. All controls are listed except binary non-response indicators for each of the above variables. Some observations with missing responses are dropped as they perfectly predicted cessation. Specifications 1 and 2 drop 7 respondents with missing flavor responses, 1 with missing

© 2020 Friedman AS et al. *JAMA Network Open.*

Hispanic ethnicity, and 1 with missing baseline education. Specifications 3 and 4 each drop 3 responses with missing race. Specification 4 also drops 1 individual with missing response to using e-cigarettes because they feel like cigarettes. Specifications 5 and 6 each drop 6 respondents missing flavor and 1 missing education response. Specifications 7 and 8 each drop 11 responses with missing flavor, 2 missing Hispanic ethnicity, 2 missing race, and 2 missing education. *(**)*** denote significance at the 0.1(0.05)0.01 levels. Linearized standard errors are adjusted for complex survey structure. Strata with a single sampling unit are treated as certainty units.

© 2020 Friedman AS et al. *JAMA Network Open.*

eTable 8. Emerging and Prime-Age Adult Cessation by Flavor, Unweighted, Adjusted Odds Ratio/Standard Error

	Cessation by Wave 3				Cessation by Wave 4			
	Emerging Adults		Prime Age Adults		Emerging Adults		Prime Age Adults	
	(1)	(2)	(3)	(4)	(5)	(6)	(7)	(8)
Vapes non-tobacco flavors	3.3033	3.3854	2.2515**	2.2535**	1.6527	1.5696	2.0149*	2.0104*
	(2.526)	(2.625)	(0.856)	(0.857)	(1.458)	(1.397)	(0.744)	(0.743)
Use e-cigarettes because		1.5189		0.9709		1.7914		1.1130
they feel like cigarettes		(0.738)		(0.300)		(1.083)		(0.351)
Male	4.7099***	5.0824***	1.8018*	1.7973*	2.3645	2.6112	1.7091*	1.7038*
	(2.462)	(2.713)	(0.568)	(0.567)	(1.437)	(1.623)	(0.551)	(0.550)
**Race/Ethnicity**								
Hispanic	0.2908	0.2640	1.1019	1.0961	1.5029	1.2836	0.9195	0.9196
	(0.254)	(0.235)	(0.685)	(0.681)	(1.266)	(1.127)	(0.656)	(0.656)
Black	1.9695	1.9179	0.7188	0.7101	5.3484*	5.6028*	0.5372	0.5467
	(1.835)	(1.793)	(0.497)	(0.493)	(5.259)	(5.508)	(0.432)	(0.440)
Other race	1.1085	1.0836	1.7430	1.7382	0.0756**	0.0567**	0.9487	0.9512
	(0.817)	(0.800)	(0.817)	(0.814)	(0.098)	(0.079)	(0.532)	(0.533)
**Household Income**								
$10,000 to $24,999	0.8298	0.8371	1.8983	1.8924	5.0743	5.5165	3.1748**	3.1216**
	(0.548)	(0.561)	(0.933)	(0.935)	(5.957)	(6.490)	(1.585)	(1.564)
$25,000 to $49,999	0.5523	0.5904	1.0317	1.0292	17.2808**	19.4068**	1.3241	1.3012
	(0.439)	(0.473)	(0.525)	(0.526)	(21.008)	(23.777)	(0.711)	(0.702)
$50,000 to $99,999	0.4182	0.4223	2.0835	2.0777	4.7454	4.8760	1.6807	1.6603
	(0.334)	(0.338)	(1.061)	(1.061)	(5.939)	(6.091)	(0.940)	(0.930)
$100,000 or more	1.7566	1.8097	4.0674**	4.0626**	55.9594***	67.5457***	4.8588***	4.7457***
	(1.767)	(1.843)	(2.278)	(2.291)	(81.548)	(99.401)	(2.867)	(2.822)
Any College	9.0767***	9.0917***	2.3016**	2.2847**	6.1698***	6.2652***	2.0005**	2.0207**
	(4.925)	(4.931)	(0.780)	(0.777)	(3.981)	(4.047)	(0.670)	(0.680)
**Baseline Age**								
35 to 44 years old			0.8835	0.8893			1.0012	0.9880
			(0.304)	(0.308)			(0.356)	(0.354)
45 to 54 years old			0.7504	0.7547			1.1283	1.1042
			(0.329)	(0.333)			(0.468)	(0.464)
Constant	0.0148***	0.0116***	0.0365***	0.0374***	0.0045***	0.0032***	0.0456***	0.0437***
	(0.014)	(0.012)	(0.023)	(0.024)	(0.007)	(0.005)	(0.028)	(0.028)
N	149	149	336	335	125	125	281	281

Notes: Unweighted logistic regressions use data from waves 1-4 of the PATH adult samples. All controls are listed except binary non-response indicators for each of the above variables. Some observations with missing responses are dropped as they perfectly predicted cessation. Specifications 1 and 2 drop 7 respondents with missing flavor responses, 1 with missing Hispanic ethnicity, and 1 with missing baseline education. Specifications 3 and 4 each drop 3 responses with missing race. Specification 4 also drops 1 individual with missing response

© 2020 Friedman AS et al. *JAMA Network Open.*

to using e-cigarettes because they feel like cigarettes. Specifications 5 and 6 each drop 6 respondents missing flavor and 1 missing education response. Specifications 7 and 8 each drop 11 responses with missing flavor, 2 missing Hispanic ethnicity, 2 missing race, and 2 missing education. *(**)*** denote significance at the 0.1(0.05)0.01 levels.

© 2020 Friedman AS et al. *JAMA Network Open.*

eTable 9. Adult Cessation by Flavor, Adjusted Odds Ratio/Standard Error

	Cessation by Wave 3		Cessation by Wave 4	
	(1)	(2)	(3)	(4)
Vapes non-tobacco flavors	2.2776**	2.2767**	1.9935*	1.9891*
	(0.903)	(0.900)	(0.755)	(0.757)
Use e-cigarettes because they feel like cigarettes		0.9747		1.1285
		(0.287)		(0.334)
Male	2.4091***	2.4026***	1.9361**	1.9316**
	(0.702)	(0.702)	(0.614)	(0.615)
**Race/Ethnicity**				
Hispanic	0.7687	0.7695	1.4034	1.3942
	(0.452)	(0.458)	(0.777)	(0.777)
Black	0.9342	0.9289	1.1660	1.1763
	(0.604)	(0.614)	(0.832)	(0.839)
Other race	1.7793	1.7716	0.7979	0.7949
	(0.830)	(0.825)	(0.515)	(0.515)
**Household Income**				
$10,000 to $24,999	1.4798	1.4787	3.0910***	3.0452***
	(0.522)	(0.511)	(1.242)	(1.212)
$25,000 to $49,999	0.7440	0.7436	2.1569*	2.1285*
	(0.271)	(0.266)	(0.860)	(0.841)
$50,000 to $99,999	1.2543	1.2544	1.7416	1.7155
	(0.596)	(0.597)	(0.800)	(0.788)
$100,000 or more	2.5964*	2.6023*	5.4046***	5.2884***
	(1.341)	(1.313)	(2.938)	(2.894)
Any College	4.2774***	4.2558***	3.0929***	3.0959***
	(1.328)	(1.328)	(0.834)	(0.835)
**Baseline Age**				
25 to 34 years old	0.7256	0.7276	1.0685	1.0553
	(0.248)	(0.246)	(0.393)	(0.396)
35 to 44 years old	0.7272	0.7318	1.0151	0.9934
	(0.232)	(0.237)	(0.421)	(0.422)
45 to 54 years old	0.8686	0.8731	1.6307	1.5779
	(0.458)	(0.476)	(0.881)	(0.909)
Constant	0.0301***	0.0306***	0.0232***	0.0224***
	(0.018)	(0.019)	(0.015)	(0.014)
N	497	496	408	408

Notes: Weighted logistic regressions use data from waves 1-4 of the PATH adult sample for respondents ages 18 to 54 at baseline. All controls are listed except binary non-response indicators for each of the above variables. Some observations with missing responses are dropped as they perfectly predicted cessation. Specification 2 drops 1 respondent with a missing response for using e-cigarettes because they felt like cigarettes as it perfectly predicted cessation. Specifications 3 and 4 each drop 17 respondents with missing flavors, 2 respondents with missing Hispanic ethnicity, and 3 respondents with missing baseline education. *(**)*** denote significance at the 0.1(0.05)0.01 levels. Linearized standard errors are adjusted for complex survey structure. Strata with a single sampling unit are treated as certainty units.

© 2020 Friedman AS et al. *JAMA Network Open.*

eTable 10. Adult Cessation by Flavor, Unweighted, Adjusted Odds Ratio/Standard Error

	Cessation by Wave 3		Cessation by Wave 4	
	(1)	(2)	(3)	(4)
Vapes non-tobacco flavors	2.3474**	2.3458**	1.8647*	1.8589*
	(0.782)	(0.782)	(0.627)	(0.626)
Use e-cigarettes because they feel like cigarettes		1.0884		1.1744
		(0.274)		(0.313)
Male	2.2712***	2.2744***	1.8341**	1.8345**
	(0.595)	(0.596)	(0.505)	(0.506)
**Race/Ethnicity**				
Hispanic	0.7067	0.6996	1.1007	1.0828
	(0.351)	(0.349)	(0.564)	(0.557)
Black	1.1036	1.1149	1.5247	1.5499
	(0.560)	(0.566)	(0.796)	(0.809)
Other race	1.4601	1.4600	0.6090	0.6027
	(0.545)	(0.545)	(0.302)	(0.300)
**Household Income**				
$10,000 to $24,999	1.4327	1.4188	3.4767***	3.4108***
	(0.533)	(0.529)	(1.560)	(1.532)
$25,000 to $49,999	0.8439	0.8360	2.2531*	2.2161*
	(0.343)	(0.340)	(1.074)	(1.058)
$50,000 to $99,999	1.4345	1.4213	2.3561*	2.3134*
	(0.590)	(0.585)	(1.178)	(1.157)
$100,000 or more	3.0813**	3.0326**	7.5174***	7.3415***
	(1.433)	(1.418)	(4.066)	(3.987)
Any College	3.4841***	3.5024***	2.7695***	2.7925***
	(0.977)	(0.985)	(0.798)	(0.805)
**Baseline Age**				
25 to 34 years old	0.9082	0.9028	1.2638	1.2498
	(0.276)	(0.275)	(0.432)	(0.428)
35 to 44 years old	0.8566	0.8466	1.1778	1.1469
	(0.296)	(0.295)	(0.441)	(0.432)
45 to 54 years old	0.7100	0.6961	1.3272	1.2677
	(0.312)	(0.308)	(0.568)	(0.552)
Constant	0.0321***	0.0311***	0.0220***	0.0209***
	(0.017)	(0.016)	(0.013)	(0.013)
N	497	496	408	408

Notes: Unweighted logistic regressions use data from waves 1-4 of the PATH adult sample, for respondents ages 18 to 54 at baseline. All controls are listed except binary non-response indicators for each of the above variables. Some observations with missing responses are dropped as they perfectly predicted cessation. Specification 2 drops 1 respondent with a missing response for using e-cigarettes because they felt like cigarettes as it perfectly predicted cessation. Specifications 3 and 4 each drop 17 respondents with missing flavors, 2 respondents with missing Hispanic ethnicity, and 3 respondents with missing baseline education. *(**)*** denote significance at the 0.1(0.05)0.01 levels.

© 2020 Friedman AS et al. *JAMA Network Open.*
